# Responsiveness of the Arabic Upper Extremity Functional Index in Patients with Upper Extremity Musculoskeletal Disorders

**DOI:** 10.3390/ijerph20054370

**Published:** 2023-02-28

**Authors:** Ali H. Alnahdi

**Affiliations:** Department of Rehabilitation Sciences, College of Applied Medical Sciences, King Saud University, P.O. Box 10219, Riyadh 11433, Saudi Arabia; alialnahdi@ksu.edu.sa; Tel.: +966-14693595; Fax: +966-14693589

**Keywords:** psychometrics, validity, upper limb, longitudinal construct validity, activity

## Abstract

The aim of this study was to examine the ability of the Arabic Upper Extremity Functional Index (UEFI) to detect change over time in upper extremity function (responsiveness) in patients with upper extremity musculoskeletal disorders. Patients receiving physical therapy care for their upper extremity musculoskeletal disorders completed the Arabic UEFI; Disabilities of the Arm, Shoulder and Hand (DASH); Numeric Pain Rating Scale (NPRS); Global Assessment of Function (GAF); and the Global Rating of Change Scale (GRC) at the initial visit and later at a follow-up assessment. Responsiveness was examined by testing predefined hypotheses regarding the correlations between the change scores in the Arabic UEFI and the other measures. The Arabic UEFI change scores demonstrated a significant positive correlation with the change in the DASH (*r* = 0.94), GAF (*r* = 0.65), NPRS (*r* = 0.63), and GRC (*r* = 0.73), which was in line with the predefined hypotheses. The Arabic UEFI change scores demonstrated a pattern of correlation with changes in other outcome measures that are consistent with the argument that the Arabic UEFI change scores represent a change in upper extremity function. The responsiveness of the Arabic UEFI was supported, and its use to monitor changes in upper extremity function in patients with upper extremity musculoskeletal disorders was supported.

## 1. Introduction

Musculoskeletal disorders represent a leading cause of disability [1], and musculoskeletal disorders in the upper extremities are prevalent disorders in the general population [2,3]. Patients with upper extremity musculoskeletal disorders report limitations in upper-extremity-related daily activities, and these limitations were perceived by the patients to be important to their functioning [4,5,6]. Examples of these patient-reported activities include, but are not limited to, doing housework, dressing, remunerative employment, driving, washing oneself, recreation and leisure, lifting and carrying objects, hand and arm use, fine hand use and changing basic body position (pushing yourself up, leaning on your hand) [4,5,6]. Based on this, using a patient-reported outcome measure (PROM) to measure upper extremity function and quantify the magnitude of limitations in these important activities is paramount to this population.

The Upper Extremity Functional Index (UEFI) is a commonly used region-specific PROM of upper extremity function that quantifies activity limitation due to upper extremity disorders encompassing the important activities to patients with upper extremity musculoskeletal disorders [4,5,6,7,8]. The UEFI demonstrated good measurement properties including internal consistency, test–retest reliability, construct validity and responsiveness [7,8,9,10,11,12]. The UEFI has been adapted into the Arabic language and its measurement properties were tested in patients with upper extremity musculoskeletal disorders and also in patients with chronic obstructive pulmonary disease [13,14]. The Arabic UEFI demonstrated excellent internal consistency, test–retest reliability, an acceptable measurement error and evidence supporting its validity as a measure of upper extremity function [13,14].

The responsiveness of the PROM refers to the ability of the PROM to detect change over time in the construct of interest [15]. For the Arabic version of the UEFI to be used clinically and in research studies to detect change over time in upper extremity function, its responsiveness has to be established. No prior studies have examined the responsiveness of the Arabic UEFI. Thus, the aim of this study was to examine the ability of the Arabic UEFI to detect change over time in upper extremity function (responsiveness) in patients with upper extremity musculoskeletal disorders. We hypothesized that the Arabic UEFI would be able to detect change over time in upper extremity function in patients with upper extremity musculoskeletal disorders.

## 2. Materials and Methods

### 2.1. Setting and Participants

Using convenience sampling, participants were recruited from three outpatient physical therapy clinics in Riyadh, Saudi Arabia. Ethical approval was obtained from the institutional review boards at the participating institutions and participants signed informed consent forms prior to participation. The study’s inclusion criteria were (1) age of at least 18 years old and (2) upper extremity musculoskeletal disorder. The study’s exclusion criteria were (1) inability to read and understand the Arabic language and (2) disorders other than upper extremity musculoskeletal disorder that were perceived by the participants as functionally limiting (spinal, neurological, cardiovascular or pulmonary disorders).

### 2.2. Procedure

The current study was designed as a prospective cohort study where participants with upper extremity musculoskeletal disorders attending physical therapy care were assessed at their initial visit (baseline assessment) and at a follow-up visit separated by at least one week. Between the testing sessions (baseline and follow up), the participants received physical therapy care that was tailored to each patient’s needs and was determined by the treating therapist alone without involvement from the research team. At the baseline assessment, the participants were asked to complete the Arabic versions of the UEFI [13]; Disabilities of the Arm, Shoulder and Hand [16]; Global Assessment of Function [17,18]; and Numeric Pain Rating Scale [19]. At the follow-up assessment, the participants were asked to complete the same outcome measures in addition to the Global Rating of Change Scale [13,20], which was used to ensure that a change in upper extremity function occurred in at least some of the participants included in the study. The outcome measures used in the current study were chosen because they measure similar and related constructs to that measured by the UEFI, are patient-reported similar to the UEFI, have evidence supporting their measurement properties and are commonly used in the literature.

### 2.3. Outcome Measures

#### 2.3.1. Upper Extremity Functional Index (UEFI)

The UEFI is a region-specific measure of upper extremity function where participants report the degree of activity limitation they experience using 20 items [7,8]. The items were scored from zero (extreme difficulty or unable to perform activity) to 4 (no difficulty), and the total score was computed by summing all the items’ scores in one total score. The total score ranged from 0 (worst function) to 80 (best function). The validity and reliability of the Arabic UEFI used in the current study was established previously [13].

#### 2.3.2. Disabilities of the Arm, Shoulder and Hand (DASH)

The DASH is a region-specific PROM that quantifies upper extremity function and symptoms [21,22]. Each of the 30 DASH items were scored from 1 (no functional limitation and no symptoms) to 5 (functional inability and extreme symptoms). The DASH total score (0–100) was obtained by multiplying 25 by the mean of the items’ scores minus one, with a higher total score indicating worse upper extremity function and symptoms [16]. There is evidence supporting the validity, reliability and responsiveness of the Arabic version of the DASH used in the current study [16].

#### 2.3.3. Numeric Pain Rating Scale (NPRS)

The average upper extremity pain intensity in the last 24 h was measured using the NPRS on a scale ranging from zero (no pain) to 10 (worst pain imaginable) [23]. The validity and reliability of the Arabic NPRS was demonstrated previously [18,19].

#### 2.3.4. Global Assessment of Function (GAF)

In the GAF, the participants rated, on a scale from 0 to 100, their ability to perform activities of daily living, with 0 indicating an inability to perform any activity of daily living and 100 indicating an ability to perform all activities of daily living without difficulty. The GAF was previously reported to be valid and reliable [18,24].

#### 2.3.5. Global Rating of Change Scale (GRC)

In the GRC, the participants rated the change in their upper extremity function at the follow up compared to the baseline assessment. An 11-point GRC (−5 to 5) was used in the current study, with a score of −5 indicating a very great deal worse, a score of 5 indicating a very great deal better and a score of 0 in the middle indicating no change [18,20,24]. The participants with GRC scores of −1 to 1 were classified in the current study as unchanged, while the participants with GRC scores of 2 to 5 and −2 to −5 were classified as improved and worsened, respectively.

### 2.4. Statistical Analysis

The ability of the Arabic UEFI to detect change over time in upper extremity function (responsiveness) in patients with upper extremity musculoskeletal disorders was examined by testing a number of predefined hypotheses regarding the expected pattern of correlation between the change scores of the Arabic UEFI and the change scores of other outcome measures [25,26]. We hypothesized that the Arabic UEFI change scores would have (1) at least a moderate positive correlation with the DASH change scores (≥0.4), (2) at least a moderate positive correlation with the GAF change scores (≥0.4), (3) at least a moderate positive correlation with the NPRS change scores (≥0.4), (4) at least a moderate positive correlation with the GRC and (5) at least a 0.1 higher absolute correlation with the DASH change scores compared to the NPRS change scores. These hypotheses were formulated a priori based on the argument that the change scores of the Arabic UEFI represent change in upper extremity function.

The change scores of the Arabic UEFI and GAF were computed as the follow-up scores minus the baseline scores while the change scores of the DASH and NPRS were computed as the baseline scores minus the follow-up scores. This was performed so that the positive change scores would indicate improved patient status (better function and less pain). The correlations between the change scores of the Arabic UEFI and the change scores of the other outcome measures were examined using Pearson’s and Spearman’s correlation coefficients. The difference between the correlation coefficients was tested using Meng’s test [27]. Differences between the baseline and follow-up scores in all the outcome measures were examined using dependent *t*-tests. The effect size (the difference between the follow-up and baseline scores divided by the standard deviation of the baseline scores) and the standardized response mean (the difference between the follow-up and baseline scores divided by the standard deviation of the change scores) were computed to quantify the magnitude of change in the Arabic UEFI and the other outcome measures [28]. The normality of the outcome measure change scores was examined via visual inspection of the histograms and using the Shapiro–Wilk test. All statistical analyses were conducted using IBM SPSS Statistics 26 (IBM Corp., Armonk, NY, USA).

#### Sample Size Estimation

The required sample size for the purpose of the current study was based on the recommendations of the consensus-based standards for the selection of health measurement instruments (COSMIN) [29]. The COSMIN considered a sample size of 50 participants to be an adequate sample size to examine the responsiveness of a PROM using hypothesis testing, which is the method used in the current study. Based on that, the minimum required sample size for the current study was determined to be 50 participants.

## 3. Results

Sixty-three participants with upper extremity musculoskeletal disorders participated in the current study (Table 1). All the participants completed the Arabic UEFI and the other outcome measures at baseline and follow-up sessions (Table 2). No missing items in the Arabic UEFI were observed for all participants at both testing sessions. The median duration between the baseline and the follow-up sessions was 25 days with a minimum time frame of 7 days and a maximum time frame of 72 days. Between the testing sessions, the participants received various combinations of physical therapy interventions that were tailored to each patient’s needs and were determined by the treating therapist with no involvement from the research team. The provided physical therapy interventions included patient education, strengthening exercises, stretching and mobility exercises, joint and soft tissue manual therapy, electrotherapy, dry needling and taping.

The majority of the participants (88.9%) reported an improvement in their functional ability compared to their baseline status based on the GRC scores at the follow-up assessment (Table 3). A minority of the participants reported either no change in their functional ability (9.5%) or a worsening functional ability (1.6%). The Arabic UEFI showed a significant increase at the follow-up (*p* < 0.001) compared to the baseline with a mean difference of 20.78 points (95% CI of the difference: 14.85–26.71) with a large effect size (Table 2). The DASH showed a significant reduction at the follow-up (*p* < 0.001) compared to the baseline with a mean difference of 26.49 points (95% CI of the difference: 19.95–33.03) with a large effect size (Table 2). The GAF showed a significant increase at the follow-up (*p* < 0.001) compared to the baseline with a mean difference of 25.60 points (95% CI of the difference: 19.63–31.57) with a large effect size (Table 2). The NPRS also showed a significant reduction at the follow-up (*p* < 0.001) compared to the baseline with a mean difference of 2.59 points (95% CI of the difference: 1.78–3.39) with a large effect size (Table 2).

**Figure 1:**
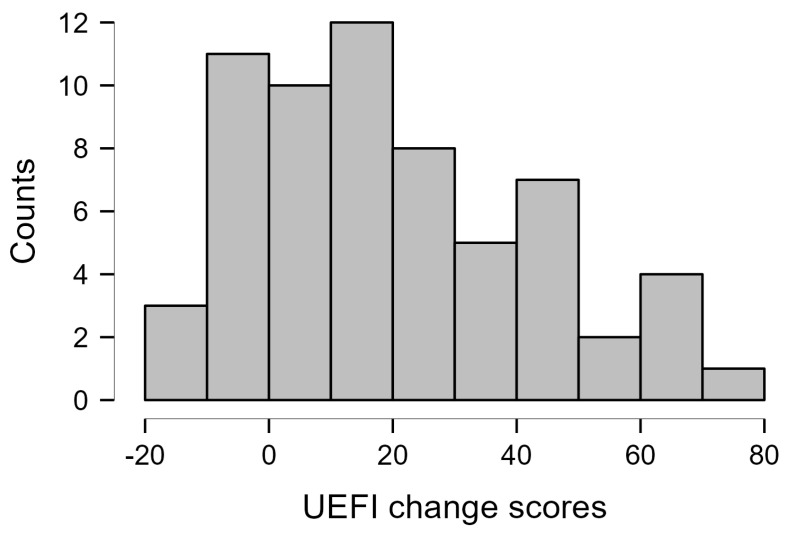
Histogram showing the Arabic UEFI change scores distribution (*N* = 63).

The Arabic UEFI change scores showed a good spread with no significant deviation from the normal distribution based on the Shapiro–Wilk test (*p* = 0.051) (Figure 1). Similarly, the change scores of the DASH, GAF and NPRS showed no significant deviation from the normal distribution (*p* = 0.27, *p* = 0.24, *p* = 0.19); thus, their correlations with the Arabic UEFI change scores were examined using Pearson’s correlation coefficient. The Arabic UEFI change scores showed a significant positive correlation above the predetermined level with the change scores of the DASH, GAF and NPRS (Table 4). The correlation between the Arabic UEFI change scores and the DASH change scores was significantly higher than the correlation between the Arabic UEFI change scores and the change scores of the NPRS (*z* = 6.61, *p* < 0.001). Given the limited spread of the participants’ GRC scores (majority indicated improved status), Spearman’s correlation was used to examine the correlation between the Arabic UEFI change scores and the GRC, and it indicated a significant positive correlation higher than the predefined level (Table 4).

## 4. Discussion

The aim of this study was to examine the ability of the Arabic UEFI to detect change over time in upper extremity function (responsiveness) in patients with upper extremity musculoskeletal disorders. In line with our hypothesis, the UEFI demonstrated a sufficient ability to detect change over time in upper extremity function in patients with upper extremity musculoskeletal disorders, which supports its use to track changes in upper extremity function over time.

Examining responsiveness require change to occur over time in the construct of interest, which was upper extremity function in this study, in at least a portion of the sample [25,29]. This was ensured by using the GRC, in which the majority of the participants indicated an improvement in their upper extremity function at the follow-up assessment compared to baseline. Additionally, the effect size and standardized response mean indicated that the magnitude of change from the baseline to follow-up was large for the Arabic UEFI and the other measures, which suggests that clinically meaningful change (better upper extremity function and less pain) occurred between the baseline and follow-up assessments [30].

The ability of the Arabic UEFI to detect change over time in upper extremity function (responsiveness) in patients with upper extremity musculoskeletal disorders was established by examining the validity of the Arabic UEFI change scores [15,25,29]. The Arabic UEFI change scores were argued to represent change in upper extremity function; thus, a number of hypotheses were formulated a priori regarding the expected pattern of correlation between the Arabic UEFI change scores and the change scores in the other outcome measures. The results of the current study supported all (100%) of our predefined hypotheses, thus support the ability of the Arabic UEFI to detect change over time in upper extremity function (responsiveness).

Given that the Arabic UEFI change scores and the change scores of the DASH and GAF represent change in the same construct (upper extremity function), at least moderate positive correlations were expected among these measures. Positive correlations were expected because higher Arabic UEFI change scores and higher change scores in the DASH and GAF as computed in the current study represent an improvement in upper extremity function; thus, they were expected to change in the same direction. The results of the current study supported both the direction and strength of the expected correlation, which supports the validity of the Arabic UEFI change scores as a measure of change in upper extremity function. In line with the findings of the current study, the UEFI change scores demonstrated a moderate-to-strong correlation with the change scores of the other measures of upper extremity function such as the upper extremity functional scale (*r* = 0.74, *r* = 0.67) [7,8], DASH (*r* = 0.90) [9] and the patient-specific functional scale (*r* = 0.63) [31] in patients with upper extremity musculoskeletal disorders. Additionally, a similar pattern of correlation was also reported between the UEFI change scores and Quick DASH change scores (*r* = 0.62) in women after breast cancer surgery [10].

The GRC is a commonly used anchor to quantify the perceived magnitude of change in clinical status at follow-up assessments compared to the baseline. The GRC used in the current study was worded to enquire specifically about change in the construct of interest, which was upper extremity function, rather than a general change in health status. Based on this, the Arabic UEFI change scores, which arguably represented change in upper extremity function, were expected to have at least moderate positive correlation with the GRC. In line with our predefined hypothesis, patients with larger Arabic UEFI change scores showed higher scores in the GRC, which supports the argument that the Arabic UEFI change scores represent change in upper extremity function. Prior literature reported a similar correlation between the UEFI change scores and the GRC. In patients with upper extremity musculoskeletal disorders, the UEFI change scores were reported to correlate moderately with GRC (*r* = 0.57) [8] (*r* = 0.51) [31]. On the contrary, one study reported a correlation between the UEFI change scores and GRC (*r* = 0.35) that was lower than the correlation observed in the current study [9]. The inclusion of patients with neck pain representing 21% of the sample might explain the difference in the magnitude of the correlation between the current study and that of Lehman and colleagues [9].

A reduction in pain intensity was hypothesized to be associated with an improvement in upper extremity function represented by the Arabic UEFI change score. This hypothesized correlation was expected to have a positive direction given that higher NPRS and Arabic UEFI change scores represent pain reduction and better function. The magnitude of the correlation was expected to be of at least moderate strength given that changes in pain intensity and changes in upper extremity function are related constructs. The results of the current study supported the hypothesized positive correlation and also supported the strength of the hypothesized correlation. Consistent with the findings of the current study, UEFI change scores were reported to correlate moderately with change scores in pain intensity measures, (*r* = 0.65) [7] and (*r* = 0.5) [8], in patients with upper extremity musculoskeletal disorders.

The change scores of the Arabic UEFI and the DASH are argued to represent change in the same construct, which is upper extremity function, while the change scores of the NPRS represent change in a related but not similar construct. Based on that, the Arabic UEFI change scores were hypothesized to show a higher correlation with the DASH change scores compared to the NPRS change scores. This hypothesized difference in the magnitude of correlation was supported by the results of the current study, which showed that the Arabic UEFI change scores had a stronger correlation with the DASH change scores compared to the NPRS change scores. In patients with upper extremity musculoskeletal disorders, the changes in the UEFI had a stronger correlation with the changes in the outcome measures that represented upper extremity function compared to the changes in measures of pain intensity, (*r* = 0.74 versus *r* = 0.65) [7] and (*r* = 0.67 versus *r* = 0.5) [8]. The UEFI change was also reported to have a higher correlation with changes in measures of upper extremity function (Quick DASH) compared to changes in pain intensity in women after breast cancer surgery [10].

The current study is not without limitations. The majority of the participants in the current study had wrist and hand problems followed by shoulder and arm problems, with elbow and forearm problems represented by a minority of the participants (14.3%). Thus, the findings of the current study should be interpreted with caution for patients with elbow and forearm musculoskeletal disorders. On the other hand, the responsiveness of the Arabic UEFI was assessed in the current study by examining specific predefined hypotheses; we used appropriate time intervals between the baseline and follow-up assessments, as well as appropriate provisions of physical therapy interventions to ensure a change in upper extremity function in an adequate number of participants.

## 5. Conclusions

The ability of the Arabic UEFI to detect change over time in upper extremity function (responsiveness) in patients with upper extremity musculoskeletal disorders was examined in this study. The Arabic UEFI change scores correlated with changes in other outcome measures in a pattern which was consistent with the argument that the Arabic UEFI change scores represent a change in upper extremity function. Thus, the responsiveness of the Arabic UEFI was supported, and its use to monitor changes in upper extremity function in patients with upper extremity musculoskeletal disorders for daily clinical practice and research studies was supported.

## Figures and Tables

**Table 1:** Characteristics of participants (*N* = 63).

Variable	Mean ± SD or *N* (%)
Age (year)	38.40 ± 13.90
Sex	
Male	38 (60.3)
Female	25 (39.7)
Height (m)	1.67 ± 0.09
Mass (Kg)	77.06 ± 18.02
Body mass index (Kg/m^2^)	27.57 ± 6.40
Site of dysfunction	
Shoulder and arm	22 (34.9)
Elbow and forearm	9 (14.3)
Wrist and hand	32 (50.8)
Upper extremity surgery	
Yes	26 (41.3)
Time after surgery (months)	1.50 (1.84) *
No	37 (58.7)
Duration of symptoms (months)	2.99 (6.79) *

* = median (interquartile range).

**Table 2:** Outcome measures at baseline and follow-up (*N* = 63).

Variable	Baseline Scores Mean ± SD	Follow-Up Scores Mean ± SD	Change Scores Mean ± SD	ES	SRM
UEFI (0–80)	42.48 ± 18.55	63.25 ± 18.21	20.78 ± 23.55	1.12	0.88
DASH (0–100)	50.01 ± 20.47	23.52 ± 23.62	26.49 ± 25.97	1.29	1.02
GAF (0–100)	56.40 ± 19.68	82.0 ± 19.36	25.60 ± 23.70	1.30	1.08
NPRS (0–10)	4.90 ± 2.61	2.32 ± 2.81	2.59 ± 3.20	0.99	0.81

UEFI = Arabic version of the Upper Extremity Functional Index; DASH = Disabilities of the Arm, Shoulder and Hand; GAF = Global Assessment of Function; NPRS = Numeric Pain Rating Scale; ES = effect size; SRM = standardized response mean.

**Table 3:** Participants according to their global rating of change score at follow-up (*N* = 63).

Variable	*N* (%)
GRC	
5 (Very great deal better)	24 (38.1)
4 (Great deal better)	14 (22.2)
3 (Moderately better)	10 (15.9)
2 (Little bit better)	8 (12.7)
1 (A tiny bit better, almost the same)	2 (3.2)
0 (No change)	2 (3.2)
−1 (Tiny bit worse, almost the same)	2 (3.2)
−2 (Little bit worse)	0 (0.0)
−3 (Moderately worse)	1 (1.6)
−4 (Great deal worse)	0 (0.0)
−5 (Very great deal worse)	0 (0.0)
Change over time status according to GRC score *	
Unchanged	6 (9.5)
Changed	57 (90.5)
Improved	56 (88.9)
Worsened	1 (1.6)

GRC = Global Rating of Change Scale. * = participants with GRC scores of −1 to 1 were classified as unchanged, while participants with GRC scores of 2 to 5 and −2 to −5 were classified as improved and worsened, respectively.

**Table 4:** Correlation between the Arabic UEFI change score and other measures (*N* = 63).

Variable	*r* (95% CI)	*p*
DASH change	0.94 (0.91 to 0.96)	<0.001
GAF change	0.65 (0.45 to 0.80)	<0.001
NPRS change	0.63 (0.46 to 0.78)	<0.001
GRC	0.73 (0.58 to 0.83) *	<0.001

*r* = Pearson’s correlation coefficient; CI = confidence interval; UEFI = Upper Extremity Functional Index; DASH = Disabilities of the Arm, Shoulder and Hand; GAF = Global Assessment of Function; NPRS = Numeric Pain Rating Scale. * = examined using Spearman’s correlation coefficient.

## Data Availability

The data presented in this study are available from the corresponding author on reasonable request.
